# Correlation of Hysteroscopy With Histopathological Findings of the Endometrium in Women With Polycystic Ovary Syndrome (PCOS)-Related Infertility

**DOI:** 10.7759/cureus.57666

**Published:** 2024-04-05

**Authors:** Kanchan Rani, Kante Durga Mounika, Shivangi Singhal

**Affiliations:** 1 Obstetrics and Gynaecology, Teerthanker Mahaveer Medical College and Research Centre, Moradabad, IND; 2 Obstetrics and Gynaecology, Teerthanker Mahaveer Medical college and Research Centre, Moradabad, IND

**Keywords:** histopathology, disordered proliferative endometrium, hysteroscopy, infertility, pcos

## Abstract

Introduction

There have been numerous studies on the anovulatory factor, leading to infertility in women with polycystic ovary syndrome (PCOS); however, studies on the endometrium factor causing infertility in PCOS women are scarce. While hysteroscopy can accurately diagnose endometrial disorders such as endometrial polyps, it may be ineffective in detecting probable endometrial pathologies due to different hormonal habitats in these patients.

Materials and methods

Sixty patients with PCOS-related infertility were included in the study. All participants underwent hysteroscopic examination followed by endometrial biopsy and histopathological examination. The clinical and hormonal profiles of two main subgroups, that is, (a) normal endometrium (N), which included proliferative endometrium and secretory endometrium on histology, and (b) disordered endometrium (D), which included disordered endometrium on histology, were compared.

Results

There was no correlation between hysteroscopic and histopathological findings of PCOS infertile women. In the subgroup analysis of the two main histological types, that is, normal (proliferative and secretory) and disordered (disordered endometrium), age (28.70±4.66 vs. 32.9±5.61, p=0.012) and duration of amenorrhea (5.49±2.43 vs. 7.82±2.93, p=0.008) were significantly higher in the disordered group. There was a statistically nonsignificant higher BMI in the patients of the disordered endometrium group.

Conclusion

These findings suggest that endometrial biopsy and histopathological evaluation along with hysteroscopy should be desired in women with PCOS-related infertility, especially if they are in the late reproductive age group and have a longer duration of amenorrhea, regardless of endometrial thickening. This approach is essential to diagnose and treat endometrial disorder, which can be an additional cause of infertility, recurrent implantation failure, and recurrent pregnancy loss, in addition to ovulatory dysfunction.

## Introduction

Polycystic ovary syndrome (PCOS) is one of the most common endocrine-metabolic disorders in females of reproductive age, with reported prevalence varying between 2.2% and 15-20% [1]. According to the International Consensus Group, for the diagnosis of PCOS, two of the following three criteria should be met: oligo/anovulation, clinical and/or biochemical hyperandrogenism, and polycystic ovarian morphology on transvaginal ultrasonography [2]. Patients with PCOS primarily present with symptoms such as irregular menstruation/oligomenorrhea, hirsutism, acne, and infertility [1]. Infertility is a major concern for women with PCOS, accounting for about 80% of anovulatory infertility cases [1]. However, even after ovulation is restored by various methods, women with PCOS continue to exhibit reduced cumulative pregnancy rates and a higher incidence of implantation failure and spontaneous abortion [3]. In vitro fertilization success rates are lower in women with PCOS, even when high-quality embryos are transferred [4]. Probable explanations include reduced progesterone levels, prolonged exposure to estrogen, higher levels of free insulin, insulin-like growth factor-1, androgens, and luteinizing hormone (LH), which can lead to aberrant endometrial cellular proliferation and receptivity [1,5]. The endocrinologic and metabolic abnormalities associated with PCOS may exacerbate endometrial diseases, potentially having complex effects on the endometrium [6]. Thus, the data suggest that both anovulation and endometrial abnormalities contribute to infertility in these women [1,2,5,7]. While numerous studies have evaluated the anovulatory factor causing infertility in women with PCOS, research on the endometrial factor causing infertility in PCOS is scarce [1].

Most studies recommend a biopsy should be performed when the endometrial thickness exceeds 12 mm [8]. However, whether an endometrial biopsy should be performed in PCOS patients without endometrial thickening on ultrasonography to assess endometrial disorders remains debatable [9].

While problems such as endometrial polyps can be accurately diagnosed with hysteroscopy and an endometrial biopsy is possible [3], several aspects of hysteroscopy remain debated [10]. Studies indicate that 20-40% of individuals with normal transvaginal ultrasonography results have mild intrauterine diseases detectable during hysteroscopy [11].

A recent study found that some infertile women with PCOS, without endometrial thickening, had normal hysteroscopic findings associated with abnormal endometrial biopsies. This raises the possibility that hysteroscopic evaluation alone might not be sufficient to detect potential endometrial pathologies in women with infertility [12].

Therefore, we conducted this study to determine whether an endometrial biopsy is required routinely in all infertile women with PCOS to detect and correct endometrial disorders. These disorders appear to be more common in women with PCOS than in those without and may not be detected by ultrasonography (USG) or hysteroscopy. They could be an additional factor causing infertility, recurrent implantation failure, and recurrent abortions in these women.

## Materials and methods

This prospective interventional study was conducted in the Obstetrics and Gynecology Department and Infertility Center of a tertiary medical college from July 2021 to June 2022. The study protocol was approved by the Institutional Research Ethics Committee at Teerthanker Mahaveer University (TMU/IEC/20-21/032). Patients were diagnosed with PCOS according to the modified Rotterdam criteria [2]. Patients were recruited based on inclusion and exclusion criteria.

Inclusion criteria included infertile women with PCOS aged 21 to 40 years with normal endometrial thickness on USG. Exclusion criteria were any other uterine lesion on USG, pelvic inflammatory disease (PID), tubal infertility, endometrial polyp on USG, and progesterone intake in the last three months.

The demographic profile, duration of infertility, type of infertility, and period of amenorrhea were noted, and the hormonal profile was evaluated. Each patient was explained the entire procedure, and written informed consent was obtained from all the patients. Patients were scheduled for hysteroscopic evaluation. Hysteroscopic findings were recorded.

An endometrial biopsy was taken from the abnormal area; if no abnormal area was noted, then the endometrial biopsy was taken from random sites under hysteroscopic guidance. Endometrial samples were sent for histopathological examination. Findings of hysteroscopic and histopathological examinations and demographic and hormonal profiles were compared using appropriate statistical tests.

Two main histologic types were classified: normal endometrium (N), which included proliferative endometrium and secretory endometrium on histology, and disordered endometrium (D), which included disordered proliferative endometrium on histology. Chronic endometritis was excluded from the disordered group for analysis because it has infective pathology and bears no correlation with the PCOS profile. Clinical parameters such as age, BMI, duration of infertility, period of amenorrhea, and hormonal profiles such as LH, FSH, LH/FSH ratio, testosterone, TSH, prolactin, and fasting blood sugar (FBS) between the two groups were compared with appropriate statistical tests.

On days 2 or 3 of a regular or artificially induced menstrual cycle, the patients' FSH and LH levels were assessed using an electrochemiluminescence immunoassay. The typical ranges are as follows: TSH ranges from 0.39 to 6.16 IU/mL, and FSH and LH range from 3.0 to 12.0 mIU/mL and 5.0 to 10.5 mIU/mL, respectively, in the follicular phase. Prolactin ranges from 1.2 to 19.5 ng/mL, and testosterone ranges from 8.40 to 48.10 ng/dL.

Parametric variables with a normal distribution were compared using an unpaired Student's t-test, and those without a normal distribution were analyzed using the Mann-Whitney U test. Categorical data were expressed using the chi-square and Fisher's exact tests. P-values of 0.05 were considered significant.

## Results

Out of 60 patients with PCOS with infertility, 53.33% were of primary infertility and 46.67% were of secondary infertility (Table 1). The majority of patients (45%) were in the 26 to 30 years age group. 66.6% were overweight and 13.33% were obese, with none of the patients being morbidly obese. Hysteroscopy revealed that 80% of the endometrium was normal, either proliferative or secretory (Table 2). And 8.33% exhibited hyperemia on hysteroscopy, and 8.33% had micropolyps on hysteroscopy, indicative of chronic endometritis. Histopathologically, proliferative endometrium was observed in 71.67% of patients, secretory endometrium in 6.67% of cases, chronic endometritis in 3.33% of cases, and disordered proliferative endometrium in 18.33% of patients. There were no cases of hyperplasia or adenocarcinoma diagnosed on histopathology (Table 3). There was no correlation between hysteroscopic and histopathological findings (Table 4).

**Table 1:** Demographic profile of patients

Patient characteristics	Number	Percentage
Age (years)		
21–25	14	23.33
26–30	27	45
31–35	9	15
36–40	10	16.67
BMI (kg/m^2^)		
Normal	12	20
Overweight	40	66.66
Obese	8	13.33
Morbidly obese	0	0
Type of infertility		
Primary	32	53.33
Secondary	28	46.67

**Table 2:** Hysteroscopic findings

Hysteroscopic findings	No. of patients	Percentage (%)
Normal	48	80
Proliferative	39	65
Secretary	9	15
Hyperplastic	2	3.33
Hyperemeic	5	8.13
Micropolyps	5	8.33
Total	60	100

**Table 3:** Histopathological findings

Histopathological findings	No. of patients	Percentage (%)
Normal	47	78.33
Proliferative	43	71.67
Secretary	4	6.67
Disordered proliferative	11	18.33
Chronic endometritis	2	3.33
Hyperplastic	0	0
Endometrial carcinoma	0	0
Total	60	100

**Table 4:** Comparison of hysteroscopic findings with histopathological findings

Hysteroscopic findings	Histopathology – normal		Histopathology – disordered		Total		Chi-square	p-value
	No.	%	No.	%	No.	%		
Hyperemic	5	10.6%	0	0.0%	5	8.6%	6.613	0.158
Hyperplastic	2	4.3%	0	0.0%	2	3.4%		
Micropolyps	3	6.4%	0	0.0%	3	5.2%		
Proliferative	28	59.6%	11	100.0%	39	67.2%		
Secretory	9	19.1%	0	0.0%	9	15.5%		
Total	47	100.0%	11	100.0%	58	100.0%		

Two cases of chronic endometritis on histopathology, associated with infective pathology, were excluded from the subgroup analysis of disordered endometrium. Among the two main histological types, normal (proliferative and secretory) and disordered (disordered endometrium), there was no significant difference in the duration of infertility between the two groups. Clinical parameters such as age (28.70±4.66 vs. 32.9±5.61, p=0.012) and duration of amenorrhea (5.49±2.43 vs. 7.82±2.93, p=0.008) were significantly higher in the disordered group. There was a statistically nonsignificant higher BMI in patients with disordered endometrium (Table 5). There were no significant differences in serum LH, serum FSH, serum testosterone, serum prolactin, and TSH levels between the two groups.

**Table 5:** Association of normal (N) and disordered (D) endometrium with age, BMI, duration of Infertility, period of amenorrhea, menstruation biopsy interval, LH, testosterone, FBS, TSH, prolactin, and FSH/LH ratio * p<0.05 is considered significant. BMI: body mass index, TSH: thyroid stimulating hormone, FSH: follicle stimulating hormone, LH: luteinizing hormone, FBS: fasting blood sugar.

Variable	Histopathology – normal (N=47)	Histopathology – disordered (N=11)	Unpaired t-test	
	Mean ± SD	Mean ± SD	t-value	p-value*
Age (years)	28.70 ± 4.66	32.91 ± 5.61	-2.59	0.012
BMI (kg/m^2^)	26.04 ± 3.09	27.99 ± 3.09	-1.89	0.064
Duration of amenorrhea (months)	5.49 ± 2.43	7.82 ± 2.93	-2.75	0.008
Duration of infertility (year)	5.55 ± 2.31	6.09 ± 2.21	-0.70	0.487
Serum TSH (µIU/mL)	3.66 ± 1.09	3.92 ± 0.93	-0.674	0.5
Serum prolactin (ng/dL)	14.53 ± 6.90	18.34 ± 9.98	-1.51	0.138
Serum FSH (mIU/mL)	5.31 ± 1.98	5.53 ± 2.53	-0.32	0.754
Serum LH (mIUm/L)	9.24 ± 3.24	9.01 ± 4.03	0.20	0.840
Testosterone (ng/dL)	43.45 ± 12.69	40.19 ± 11.02	0.79	0.435
FBS (mg/dL)	91.74 ± 7.33	90.91 ± 7.73	0.34	0.737
LH/FSH ratio	1.88 ± 0.71	1.76 ± 0.74	0.50	0.621

## Discussion

PCOS is one of the major causes contributing to infertility [13]. Studies have mainly focused on anovulation and ovarian dysfunction in PCOS infertile women [14]. However, endometrial factors are crucial for fertility [15]; as a result, endometrial histology assessment is a crucial tool for detecting endometrial problems in PCOS-affected women. Despite the fact that hysteroscopy has high patient acceptability [3] and can be used to identify endometrial abnormalities including endometrial polyps and assist targeted endometrial biopsies [10,11], several aspects of the procedure are still debatable [4]. Patients with normal transvaginal ultrasonography may have a 20-40% prevalence of mild intrauterine diseases during hysteroscopy [10,16]. In a large retrospective research including 1500 women who had diagnostic hysteroscopy, Garuti and colleagues found that hysteroscopy had the best sensitivity and specificity for diagnosing endometrial polyps and the lowest for diagnosing endometrial hyperplasia [17].

Consequently, to rule out endometrial illness, do all PCOS infertile individuals need to undergo an endometrial biopsy remains an unresolved question. The endometrial thickness on transvaginal ultrasound examination in postmenopausal women with uterine bleeding has been positively connected with the presence of endometrial abnormalities, and biopsy is recommended if the endometrial thickness is greater than 4 mm [12,18]. Unfortunately, there are no precise clinical guidelines for PCOS patients on when an endometrial biopsy should be performed, especially in infertile women to rule out endometrial disease [19].

In our study, we investigated women with PCOS whose transvaginal ultrasonography revealed appropriate endometrial thickness; hysteroscopic endometrial biopsies revealed an assortment of endometrial lesions, including proliferative endometrium (71.67%), disordered proliferative (18.33%), chronic endometritis (3.33%), and secretory endometrium (6.67%) in histopathology. The high prevalence of endometrial disorders, that is disordered proliferative endometrium in PCOS infertile patients with normal endometrial thickness on ultrasonography, in this study is an important finding considering the fact that previous studies have investigated patients with increased endometrial thickness in PCOS women, reporting different rates of endometrial disease [3,4].

Disordered proliferative endometrium is abnormal proliferative endometrium with architectural changes due to persistent unopposed estrogen stimulation, considered benign, not precancerous [20,21].

It is common in patients with polycystic ovarian syndrome, obesity, and perimenopausal women associated with anovulation and can be treated with progesterone [22]. Unopposed estrogen on disordered proliferative endometrium in the early phase can later develop into hyperplasia [23].

In a similar study by Amooee et al., they found 17.1% disordered proliferative endometrium in PCOS infertile women with normal endometrial thickness on ultrasound and normal hysteroscopic findings, which corresponds to our study results. They also showed that there was no correlation between hysteroscopic and histological results (p = 0.28, contingency coefficient = 0.3) [12].

In the present study, we found age and the period of amenorrhea were significantly higher in the disordered proliferative endometrium group (28.70±4.66 vs. 32.91±5.61, p=0.012 and 5.49±2.43 vs. 7.82±2.93, p=0.008). This was similar to a study by Park et al., where they found that compared to women with proliferative endometrium, those with endometrial illness had a significantly higher mean age in the PCOS group (28.26±6.80 years vs. 25.10±5.37 years; p=0.013) [24]. In contrast, Amooee et al. did not find any correlation between age and endometrial disorder in PCOS women [12].

Cheung [21] showed that an endometrial thickness higher than 7 mm or an intermenstrual interval longer than 3 months can be indicators of endometrial hyperplasia in infertile PCOS women, and an endometrial biopsy is advised. Other authors claimed that even when the endometrial thickness is normal (5-12 mm), an endometrial biopsy is necessary when the patient has a clinical history that points to long-term, unopposed estrogen exposure, and that an endometrial biopsy is necessary regardless of the patient's clinical history when the endometrial thickness exceeds 12 mm [5].

We found no association of serum LH, serum FSH, LH/FSH ratio, serum testosterone, TSH, serum prolactin, and fasting blood sugar with disordered proliferative endometrium, although higher quality assays like liquid chromatography-mass spectrometry, extraction/chromatography immunoassays, and high-quality radioimmunoassays are more accurate for serum testosterone but less readily available. Similarly, immunohistochemistry is more sensitive for serum LH but it is less affordable and less widely available.

The BMI of patients in the disordered proliferative group in our study was nonsignificantly higher than in the histopathologically normal group (26.04±3.09 vs. 27.99±3.09, p=0.064). Previous studies have also shown a correlation of obesity with disordered proliferative endometrium [22].

Our study revealed no premalignant or malignant lesions despite the presence of various endometrial disorders. Nonetheless, these findings underscore the importance of assessing endometrial abnormalities in PCOS infertile women, further to its established relationship with endometrial thickness [25].

Endometrial micropolyps are small lesions (1-2 mm in length) that can only be detected on hysteroscopy [26]. According to retrospective research, endometrial micropolyps were assessed to be 11% common during hysteroscopy using traditional tissue staining, and they were linked to endometritis and infertility [26]. Chronic endometritis, characterized by plasma cell infiltration in the uterine stroma, endometrial stromal edema, and periglandular hyperemia, often accompanies endometrial micropolyps [27].

In our study, 8.33% of individuals with PCOS had micropolyps, even though only 3.33% of plasma cells were identified in histopathology to diagnose endometritis. The lower rate of detection of chronic endometritis may be due to the difficulty, even for experienced pathologists, in distinguishing stromal plasma cells from stromal fibroblasts and mononuclear leukocytes in the endometrium by conventional tissue staining (e.g., methyl green-pyronin and hematoxylin-eosin). This difficulty arises because stromal plasma cells in the endometrium share many histological characteristics with these cells [28]. To diagnose endometritis, supplementary diagnostic methods, including immunohistochemistry, have been suggested [29]. Infertility and endometrial hyperplasia/cancer are associated with chronic endometritis and micropolyps, as are the effects of therapy on these conditions [30].

Since there was no correlation between hysteroscopic and histological results in the current study, it is possible that normal hysteroscopic findings may not necessarily imply a normal endometrium, and that histopathology is necessary, particularly in PCOS-affected women who may not have thick endometrium. Despite the fact that no premalignant or malignant endometrial lesions were found in the current investigation, hysteroscopy without biopsy was unable to detect most chronic endometritis patients and endometrial proliferative diseases.

This study revealed the endometrial abnormality of women with PCOS having normal endometrial thickness on vaginal ultrasonography. In regard to endocrinology, the endometrium of women with PCOS appears to be different from that of healthy women. The results showed that in most of the infertile PCOS women, the hysteroscopic results were normal, but the histology findings were different. These findings reflect that direct hysteroscopy visualization, the gold standard for the detection and treatment of intrauterine abnormalities and infertility, may not be adequate to detect endometrial abnormalities in PCOS-affected women with infertility who have different hormonal milieu from other patients.

## Conclusions

These findings suggest that endometrial biopsy and histopathological evaluation, along with hysteroscopy, should be desired in PCOS infertile women, especially if they are in the late reproductive age group and have a longer duration of amenorrhea, regardless of endometrial thickening, in order to diagnose and treat endometrial disorder. This condition can be an additional cause of infertility, recurrent implantation failure, and recurrent pregnancy loss, in addition to ovulatory dysfunction. Age, duration of amenorrhea, and BMI may be associated with disordered endometrium, but larger studies are necessary to determine the optimum cutoff values.
